# The Relationship between Breastfeeding and Initial Vegetable Introduction with Vegetable Consumption in a National Cohort of Children Ages 1–5 Years from Low-Income Households

**DOI:** 10.3390/nu14091740

**Published:** 2022-04-22

**Authors:** Hannah R. Thompson, Christine Borger, Courtney Paolicelli, Shannon E. Whaley, Amanda Reat, Lorrene Ritchie

**Affiliations:** 1 Community Health Sciences, School of Public Health, University of California, 2121 Berkeley Way, 6120, Berkeley, CA 94720, USA; 2 Nutrition Policy Institute, Division of Agriculture and Natural Resources, University of California, 1111 Franklin Street, Fifth Floor, Oakland, CA 94706, USA; lritchie@ucanr.edu; 3 Westat, 1600 Research Boulevard, Rockville, MD 20850, USA; christineborger@westat.com; 4 USDA Food and Nutrition Service, 1320 Braddock Place, Alexandria, VA 22314, USA; courtney.paolicelli2@usda.gov (C.P.); amanda.reat@usda.gov (A.R.); 5 Public Health Foundation Enterprises WIC, Division of Research and Evaluation, 12781 Schabarum Avenue, Irwindale, CA 91706, USA; shannon@phfewic.org

**Keywords:** vegetable consumption, vegetable variety, breastfeeding, timing of vegetable introduction, complementary feeding

## Abstract

Compared to other food groups, vegetable intakes are lowest relative to recommendations. Breastfeeding and initial introduction to vegetables may help infants establish long-lasting taste preferences. We examined the relationship between breastfeeding and initial vegetable introduction and vegetable intake in early childhood (ages 13–60 months). This repeated cross-sectional study used data from the national WIC Infant and Toddler Feeding Practices Study-2 collected from low-income mother/caregivers about infants from around birth through age 5 (60 months; n = 3773). Survey-weighted adjusted regression models assessed associations between breastfeeding and vegetable introduction measures with vegetable consumption at child ages 13, 24, 36, 48, and 60 months. Longer breastfeeding duration was associated with a slightly, but significantly, greater variety of vegetables consumed/day in early childhood. There was also a small but positive statistically significant association between the number of different types of vegetables consumed on a given day at 9 months and the amount and variety of vegetables consumed/day in early childhood. Age of initial vegetables introduction and whether vegetables were the first/second food introduced were not consistently related to the amount or variety of vegetables consumed later in childhood. Longer breastfeeding and introduction to a greater variety of vegetables at 9 months may be behaviors to target to increase consumption of a greater variety of vegetables by young children.

## 1. Introduction

Eating a diet rich in vegetables is linked to the prevention of numerous poor health outcomes such as obesity, cardiovascular disease, and certain cancers [1]. Evidence demonstrates that consumption of a greater variety of vegetables is also related to better health [2,3,4,5]. The 2020–2025 Dietary Guidelines for Americans, therefore, includes recommendations for total vegetables as well as for subgroups: dark green, red and orange, legumes, starchy, and other vegetables [5]. However, compared to other major food groups, children’s vegetable intakes rank the lowest relative to recommendations [5,6]. On a given day, over a quarter of young children in the United States do not consume any vegetables [7], and consumption is typically lower for children living in low-income households [8,9]. Moreover, vegetable intakes decline after 1 year of age, as children tend to become pickier and exert more eating autonomy [10,11,12].

One reason for young children’s reluctance to eat vegetables is that some are naturally more bitter and less sweet than preferred foods. Children are born with a predilection for sweet tastes and an aversion to bitter tastes [13]. These early preferences, however, are not immutable [14]. Studies have shown that children more readily accept the taste of vegetables and continue to eat them later in life if given exposure early and often [9,15].

Even before consumption of solid foods, infants are exposed to flavor cues in utero and postnatally through breastmilk [16]. A recent review concluded that there is moderate evidence that plant-based flavors from a mother’s diet are detectible by infants at 1–4 months postpartum [17]. Multiple studies suggest that breastfeeding, compared to formula feeding, or longer breastfeeding duration are associated with greater vegetable consumption by children [18,19,20]. While rates of breastfeeding have increased in the United States in recent decades [21], they remain lower among low-income women compared to the national average [22].

Introduction to vegetables when developmentally appropriate (e.g., at around 6 months) and continuing during the first year of life may help to establish taste preferences [9]. Infancy is viewed as an especially malleable period for setting the stage for lifelong eating habits [23]. However, while preferences for vegetables have been shown to track from ages 2–3 years through later childhood [24,25], few studies have examined the impact of infant feeding practices on vegetable intakes later in childhood. For example, in a comprehensive review [26], only one study examined longer term impacts of complementary feeding of vegetables, finding that introducing vegetables before fruit to infants increased vegetable intake at 12 months of age, a difference that was no longer detectible at 23 months [27].

Further, more evidence is needed on how best to introduce and promote vegetable intakes among young children, particularly those from low-income families, who historically have faced greater barriers to adequate vegetable intake [9,26]. Understanding the relationships between breastfeeding and the timing of introduction to vegetables in the first years of life and vegetable consumption in early childhood can help inform infant feeding recommendations in the Dietary Guidelines for Americans, with a long-term goal of increasing vegetable consumption during childhood and improving health outcomes.

The purpose of this study was to examine the association between breastfeeding duration and initial introduction to vegetables with subsequent dietary intake of vegetables at 1-5 years of age using data from a national cohort of children from low-income families enrolled around the time of birth in the Special Supplemental Nutrition Program for Women, Infants and Children (WIC).

## 2. Materials and Methods

### 2.1. Design

This was a secondary analysis using data from the WIC Infant and Toddler Feeding Practices Study-2 (WIC ITFPS-2). WIC ITFPS-2 is a national, longitudinal examination designed to characterize feeding practices employed by mothers/caregivers and the nutrition and health outcomes of children through 9 years of age who enrolled in WIC around birth [28,29]. The study was approved by the Westat Institutional Review Board (IRB) and state and local IRBs as needed. The WIC ITFPS-2 study is registered at ClinicalTrials.gov as NCT02031978.

### 2.2. Setting and Sample

The current study’s sample involves participants from WIC ITFPS-2 using the data available through child age 5 years. Administered by the Food and Nutrition Service at the United States Department of Agriculture (USDA), WIC provides supplemental foods, health care referrals, and nutrition education to eligible pregnant and postpartum women and children up to the month a child turns 5 years old. WIC also provides breastfeeding support and education to participants who choose to breastfeed. To qualify for the program, households must meet income eligibility guidelines and program participants must also be at nutritional risk. From July–November 2013, WIC ITFPS-2 study staff recruited participants in person from 80 WIC sites across 27 US states and territories using two-staged stratified sampling [29,30]. Eligibility criteria included being at a WIC site expected to enroll at least 30 new study-eligible cases per month (which included 37% of WIC sites and 87% of WIC participants nationally); being at least 16 years of age; being an English or Spanish speaker; and enrolling in WIC for the first time for that pregnancy or for an infant under 2.5 months old.

WIC ITFPS-2 participants (mother/caregiver–child dyads) were enrolled in either a core longitudinal sample or a supplemental sample [29], and the current analysis includes participants with data collected at ages 1, 3, 5, 7, 11, 13, 15, 18, 24, 30, 36, 42, 48, 54, and 60 months (see Supplementary Table S1). Interview windows typically spanned +/− 2 weeks around the interview date (for example, the 6-month interview window was from child age 5.5 months to 6.5 months). Of note, the study continued to follow participants through 60 months, even if they were no longer enrolled in WIC. Informed consent was obtained from all subjects involved in the study. Mothers/caregivers received a $50 incentive for enrolling in the study; incentives ranged from $20 for additional interviews in the first two years, up to $60 by the 60-month interview.

### 2.3. Dietary Intake

Mother/caregivers completed at least one single 24 h dietary recall for the study child at 1, 3, 5, 7, 9, 11, 13, 15, 18, 24, 36, 48, and 60 months. These dietary recalls were administered via telephone by trained interviewees using the USDA’s Automated Multiple-Pass Method [31]. Prior to interviews, participants received a mailed package including measuring guides to help them report their child’s portion sizes. Food and beverages consumed were converted to food and nutrient intakes using the USDA’s Food and Nutrient Database for Dietary Studies [32]. Data were used to estimate each child’s dietary intake on a given day (using one day of recall) [33].

Vegetable consumption. Food pattern equivalent values were appended to the dietary recall data at each interview timepoint using the Food Pattern Equivalents Database (FPED) [34] to determine daily cup equivalent intake of: (1) dark green vegetables (e.g., kale, spinach, broccoli); (2) tomatoes and tomato products; (3) other red and orange vegetables, excluding tomatoes and tomato products (e.g., carrots, pumpkin, sweet potatoes); (4) white potatoes (e.g., baked, boiled, mashed, or fried, excluding potato chips); (5) other starchy vegetables, excluding white potatoes (e.g., green peas, cooked corn, lima beans); (6) legumes (beans and peas computed as vegetables); and (7) other vegetables not included in the vegetable components listed above (e.g., cucumber, celery, asparagus, avocado, onions, baby food vegetables). Total vegetables were calculated as the sum of vegetable types 1–7.

To determine the number of different vegetable types consumed on a given day, an additive score (0–7) was calculated for each dietary intake interview, with consumption (>0 cup equivalents) of dark green vegetables, tomatoes and tomato products, other red and orange vegetables, white potatoes, other starchy vegetables, other vegetables, and legumes, each counting as 1 point.

Age at introduction to vegetables was defined as the child’s age at which the mother/caregiver first reported the child consumed vegetables. To determine timing of initial vegetable introduction relative to other foods (i.e., were vegetables the first, second, etc., food introduced), we examined the timing of vegetable introduction relative to the timing of introduction to 14 other food/beverage categories (water, soda and other sugar-sweetened beverages, 100% fruit juice, tea/broth, cow’s milk, other dairy products, eggs, meats, baby cereal, other cereal, fruit, peanut butter, salty snacks, and sweet snacks; not including infant formula) up to 24 months of age as reported by mother/caregiver in response to interview questions. For example, if a mother/caregiver reported her child was first introduced to vegetables at 9 months and was also introduced to eggs and fruit at that same age, vegetables, eggs, and fruit all received an introduction ranking of “first” (i.e., rankings were not mutually exclusive). A dichotomous variable was created and coded as 1 if vegetables were among the first or second foods introduced and coded as 0 if vegetables were introduced among the third, fourth, fifth, etc., or if they were not introduced at all before age 24 months.

Breastfeeding. To assess breastfeeding duration, at each postnatal interview through 13 months, the mother/caregiver was asked if she was feeding her child human milk. During the first interview in which a mother/caregiver reported no longer feeding any human milk, she was asked how old her child was when she completely stopped feeding human milk. Breastfeeding duration in days was calculated based on this response and converted to duration in months. If a mother/caregiver was still breastfeeding at her 13-month interview, her breastfeeding duration was censored to the date of that interview. Mothers/caregivers who were not breastfeeding when discharged from the hospital post-birth were set to a breastfeeding duration of 0 days (6% of infant participants received some breastfeeding in the hospital but were coded as “not breastfed after hospital discharge” because they stopped breastfeeding at hospital discharge). Dichotomous variables were created to indicate if a child received any breastmilk at 3, 6, and 12 months.

### 2.4. Demographic Measures

Mother/caregiver race and ethnicity (Hispanic/non-Hispanic, Black/non-Hispanic, White/other) and study child date of birth and sex were collected from the mother/caregiver at the baseline interview. Time-varying mother/caregiver sociodemographic characteristics, including education level (high school or less/more than high school) and marital status (married/unmarried); household characteristics, including household federal poverty level (FPL) (at or below 75% FPL/75-130% FPL/above 130% FPL) and household food security (very low food security/ low food security/ high or marginal food security) [35]; and child WIC participation status (WIC participant/ WIC non-participant) were self-reported during follow-up participant interviews (see Table 1). If a particular demographic characteristic was not reported during a specific follow-up interview, the prior interview report was used (i.e., if marital status was missing at child age 48 months, marital status at child age 36 months was used).

### 2.5. Data Analysis

Descriptive statistics were used to characterize the study population, with and without statistical weights, at each outcome timepoint of interest (13, 24, 36, 48, and 60 months). For the primary analytic models, outcome variables included: (1) total vegetable intake in cup equivalents/day, (2) the number of different types of vegetables consumed at 13, 24, 36, 48, and 60 months, and (3) daily cup equivalent intake of dark green vegetables, tomatoes and tomato products, other red and orange vegetables, white potatoes, other starchy vegetables, legumes, and other vegetables (each as a separate outcome). Predictors included: (1) infant breastfeeding (not breastfed after hospital discharge; any breastfeeding at 3 months; any breastfeeding at 6 months; any breastfeeding at 12 months; and breastfeeding duration in months) and (2) timing of vegetable introduction (age in months when vegetables were first introduced; number of different types of vegetables eaten at nine months; vegetables were first or second food introduced). To determine associations between outcomes and predictors, we ran multiple repeated cross-sectional linear (for continuous outcomes) and logistic (for binary outcomes) regression models. All models were adjusted for child sex and WIC participation status, maternal/caregiver race and ethnicity, and time-varying maternal/caregiver- (marital status, education) and household- (federal poverty level and food security) level demographic characteristics. Models with infant breastfeeding as a primary predictor also adjusted for age of introduction to vegetables; models with timing of vegetable introduction as primary predictor also adjusted for infant continuous breastfeeding duration in months.

In analyses, data were weighted using either cross-sectional or longitudinal survey weight as appropriate to represent the national population of study-eligible infants. Sampling weights accounted for unequal sampling rates and potential nonresponse bias. Participant characteristics (poverty; maternal weight status, age, ethnicity, language preference and race; timing of WIC enrollment, child sex) used in non-response adjustment were selected for associations with response probability and study outcomes. Weighting procedures have been detailed previously [28]. All analyses were calculated using Stata MP, version 16.1 (StataCorp LP, College Station, TX, USA) [36] with results considered statistically significant at *p* < 0.05.

## 3. Results

Weighted characteristics of WIC ITFPS-2 participants included in this analysis are presented in Table 1; unweighted characteristics (n = 3773) can be found in Supplementary Table S2. Based on the weighted sample, at age 13 months, 85.9% of study sample participants were enrolled in WIC; this declined to 52.7% by 60 months. The majority of mother/caregivers identified as Hispanic (46.3%), followed by non-Hispanic White (28.1%) and non-Hispanic Black (19.7%). At 13 months, nearly one third (62.3%) of mothers/caregivers had a high school education or less and were unmarried (63.1%); most (52.9%) lived in households at or below 75% of the FPL and experienced high or marginal food security (67.4%).

Based on the 13-month weighted sample, breastfeeding exposure declined as infants got older: 24% of child participants were not breastfed after hospital discharge; 61.4% had any breastmilk at 3 months; 27.8% any breastfeeding at 6 months; and 18.2% any breastfeeding at 12 months. On average, infants were first introduced to vegetables at 5.7 months (SD ± 0.05) and by 9 months were consuming an average of 1.2 different types of vegetables per day (SD ± 0.04). A small proportion of parent/caregivers (4.5%) reported introducing vegetables before 4 months of age. For over three-quarters (76.2%) of children, vegetables were among either the first or second foods introduced. At 13 months, average total vegetable intake was 0.6 cup equivalents/day (SD ± 0.01) and children were consuming a mean 2.2 different types of vegetables/day (SD ± 0.04).

In examining the association between breastfeeding exposure and timing of vegetable introduction with total daily vegetable intake, we found children who were not breastfed after hospital discharge reported slightly lower total vegetable consumption at 60 months, compared with children who were breastfed (−0.08 cup equivalents/day; Table 2). Children who had any breastfeeding at 3 months reported slightly greater vegetable consumption at 60 months (0.14 cup equivalents/day; the equivalent of one additional cup of vegetables/week) compared to children with no breastfeeding at 3 months. Not breastfeeding after hospital discharge or any breastfeeding at 3 months was not associated with total vegetable consumption at 13, 24, 36 or 48 months. Any breastfeeding at 6 months, any breastfeeding at 12 months, and breastfeeding duration were not associated with total vegetable consumption at any age.

The number of different types/day of vegetables consumed on a given day at 9 months of age was positively and consistently associated with total vegetable consumption in early childhood; for every increase in the number/day of different types of vegetables consumed at 9 months, intake of total vegetable cup equivalents/day increased at ages 13 (0.06), 24 (0.05), 36 (0.04), 48 (0.05) and 60 (0.09) months (Table 2; note that the point estimate at 48 months, while consistent with findings at the other ages, is not statistically significant). Children whose first or second food consumed was a vegetable reported significantly higher total vegetable intake (0.14 cup equivalents/day) at 13 months only.

We additionally examined the association between breastfeeding exposure and timing of vegetable introduction with daily intake of specific vegetable types (see Supplementary Table S3). Compared with children who were not breastfed after hospital discharge, children who were not breastfed reported significantly lower consumption at 60 months of dark green vegetables (−0.04 cup equivalents/day), other red and orange vegetables (excluding tomatoes and tomato products) (−0.02 cup equivalents/day), and other vegetables (−0.06 cup equivalents/day). Children who had any breastfeeding at 3 months reported greater consumption at 60 months of other vegetables (0.10 cup equivalents/day) compared to children with no breastfeeding at 3 months. The number of different types of vegetables consumed at 9 months was positively associated with intake at 60 months of other red and orange vegetables (excluding tomatoes and tomato products) (0.02 cup equivalents/day), and other vegetables (0.03 cup equivalents/day).

In examining the association between breastfeeding exposure and timing of vegetable introduction with the number of different types/day of vegetables consumed, children who were not breastfed after hospital discharge reported a significantly lower number of different types of vegetables consumed at 60 months, compared with children who were breastfed for any duration after hospital discharge (−0.10 types/day; Table 3). Children who had any breastfeeding at 3 months, 6 months, and 12 months consistently reported a greater number of types of vegetables consumed at 13 (range 0.10–0.11 types/day), 24 (0.12–0.22 types/day), 48 (0.13–0.14 types/day), and 60 (0.15–0.24 types/day) months compared to children with no breastfeeding at 3, 6, or 12 months. There were no associations between breastfeeding exposure and number of different types of vegetables consumed at 36 months. Breastfeeding duration was also associated with a greater number of types of vegetables consumed (0.01 types/day) at 13, 24, 48, and 60 months.

For every one-month increase in the age at which vegetables were introduced, there was a slight decline in the number of different types/day of vegetables consumed at 13 (−0.03 types/day) and 60 months (−0.03 types/day) only. The number of different types/day of vegetables consumed at 9 months was positively and consistently associated with the number of different types/day of vegetables consumed in early childhood; for every increase in the number of different types/day of vegetables consumed at 9 months, the number of different types/day of vegetables consumed increased at ages 13 (0.09), 24 (0.08), 36 (0.06), 48 (0.06) and 60 (0.07) months (Table 3). There were no associations between whether vegetables were the first or second food introduced and the number of different types/day of vegetables consumed at any age.

## 4. Discussion

In a national cohort of children from low-income households, we found positive associations between the variety of vegetables consumed (as measured by the number of different types reported on a given day) at 9 months of age and both total daily vegetable intake and the number of different types of vegetables consumed per day in early childhood. While we cannot infer causality from this observational study, these findings suggest that early exposure to a greater variety of vegetable types in infancy may be beneficial. Our study provides longer-term support of findings from short trials showing that exposure to a variety of vegetables over periods ranging from a week to a month result in greater acceptance of vegetables by infants [23,37]. Future research should explore the impact of exposure to vegetable variety in infancy, particularly as it relates to changes in vegetable availability as part of the WIC food package, on total vegetable intakes during the school-age and adolescent years.

Consistent associations between infant breastfeeding and total daily vegetable intake were not apparent in the WIC ITFPS-2 sample. Children who were not breastfed after hospital discharge reported significantly lower total vegetable consumption only at 60 months, and children who had any breastfeeding at 3 months reported greater vegetable consumption only at only 60 months. Any breastfeeding at 6 months or 12 months, and breastfeeding duration as a continuous variable were not associated with total vegetable consumption at any age. It is possible that exposure to breastmilk and the flavors imparted from the maternal diet in the first several months of life are more influential on diet in infancy than in later childhood. Mennella et al. [37] conducted a randomized control trial of the impact of different timing and duration of exposure of breastfeeding moms to several vegetable juices. They found that the timing, but not the duration (from 1 to 3 months), was related to infants’ responses: infants whose mothers consumed the juices during the first month postpartum had a greater preference for carrot-flavored cereal than infants whose mother consumed the juices later postpartum. Further, in the present study, we did not look at breastfeeding intensity or exclusivity, which could impact these findings; Perrine et al. [20] found that infants exclusively breastfed for at least 3 months or more had a greater odd of consuming more than the median daily frequency of vegetables at 6 years.

Why associations would be observed between breastfeeding and child outcomes, particularly total vegetable intake, at 60 months and not earlier in childhood is unclear. One would expect that any impact of infant feeding practices would diminish as children get older and are exposed to more environmental cues and foods. All seven of the prior observational studies included in a recent review [26] found positive associations between a measure of breastfeeding (any breastfeeding duration, exclusive breastfeeding duration, or any breastfeeding versus none) and subsequent child dietary intake of vegetables measured at up to 6 years of age. To our knowledge, this is the only observational study to focus exclusively on children who were from low-income families enrolled in WIC as infants. Lower socioeconomic position has been associated with both shorter duration of breastfeeding [38] and lower vegetable intake by children [39]. While we, and all prior studies included in the recent review, adjusted for measures of maternal income and education, it is possible that residual confounding could impact our findings, as we were unable to adequately adjust for all family or environmental influences, including differences in maternal diet. Further, we used a measure of breastfeeding duration, but not intensity or exclusivity of breastmilk feeding.

While not associated with total vegetable consumption in early childhood, we did find that breastfeeding duration was positively associated with the number of different types (variety) of vegetables consumed by children from 13 to 60 months of age. Although more is known about the relationship between breastfeeding and amount, compared to variety, of vegetable intake of young children, several studies have found that a longer duration of breastfeeding is related to a greater vegetable variety of infants or toddlers [40,41]. We extend these findings by showing longer-term impacts up to an age of 5 years old.

Consistent with the American Academy of Pediatrics, WIC recommends that depending on an infant’s developmental readiness, complementary foods be introduced around 6 months of age and not before 4 months [6]. There is some evidence that introducing foods before 6 months of age is associated with food neophobia and limited dietary variety among preschool-age children [42]. On the other hand, Coulthard et al. [43] found that introduction to more textured foods after 9 months of age (compared to between 6 and 9 months) was associated with intake of less quantity and variety of vegetables by children at age 7 years. Taken together, these studies suggest that either earlier or later than recommended exposure to complementary foods may be detrimental. However, similar to other cohort studies [11,44,45], we did not find consistent associations between the timing of the initial introduction to vegetables (at 5.6 months on average) with subsequent amount or variety of vegetables consumed in early childhood. The majority of children in our sample (75%) were introduced to solid foods between 4 and 7 months of age, which may have limited our ability to detect a relationship between timing of introduction and vegetable intake outcomes.

WIC recommends that the first foods introduced to infants be either iron-fortified cereal or meat (for adequate iron and zinc), with other foods gradually introduced in no specified order thereafter [6]. In our sample, on average, 75% of participants had vegetables/legumes among the first food introduced; among those who were not first introduced to vegetables/legumes, most participants had baby cereal, fruit juice, or fruits as the first food(s) introduced. We did not find consistent associations between whether vegetables were introduced first or second (before other complementary foods) with subsequent amounts or variety of vegetables consumed in early childhood. A previous study [26] found that vegetable intake at 12 months was higher among infants who had been introduced to vegetables for the first 2 weeks of complementary feeding; however, these differences were no longer apparent when the children were 23 months old.

To our knowledge, this is the first study to examine early feeding practices in relation to type of vegetables consumed in subsequent toddler and preschool years. Compared to infants with exposure to any breastfeeding, infants who were not breastfed after hospital discharge had lower intakes of dark green, other red and orange, and other vegetables at age 60 months (5 years), while no differences were observed in intake of tomatoes, white potatoes, or legumes. Children introduced to a greater number of different types of vegetables by 9 months of age, consumed more other red and orange and other vegetables at 60 months. It is possible that we observed no associations with tomatoes and potatoes as these foods and the forms in which they are commonly consumed are readily accepted by most children; our previous work with this sample demonstrated that the top 2 vegetables, each consumed by nearly 20% of WIC ITFPS-2 participants at 48 months, were tomatoes and fried potatoes [28]. However, it has been hypothesized that exposure to the flavors from bitter compared to sweeter vegetables in breastmilk may be less impactful on children’s subsequent liking of vegetables [46]. In the randomized controlled trial by Mennella [47], preference for carrots, a relatively sweet vegetable, increased after mothers consumed increased amounts of either sweet (carrot, beet) or bitter (celery and mixed root) vegetable juices, but not for broccoli, a bitter vegetable. In a study of longitudinal cohorts in four European countries with different cultural dietary patterns, breastfeeding duration was significantly related to intake of vegetables by children at ages 2–4 years in the UK and France, but not in Portugal and Greece [18]. Whether the types of vegetables mothers consume while breastfeeding has differential impacts on infants, or whether infant exposure to breastmilk or vegetables during infancy impact preferences for some vegetables more than others, merits further study.

Several limitations deserve mention. First, this study is limited by its observational design, which precludes drawing causal inference. Second, while the USDA’s Automated Multiple-Pass Method [31] is considered a gold standard for 24 h dietary recalls [48], findings may be biased due to incomplete caregiver reporting of child intake. Although caregivers were interviewed up to 6 times during the infant year, they also may have had imperfect reporting of breastfeeding practices and timing of introduction to vegetables. Third, we did not assess maternal intake of vegetables, which is associated with children’s vegetable intake [26,49]. Fourth, we used a measure of breastfeeding duration, but not intensity or exclusivity of breastmilk feeding. Fifth, we acknowledge that effect sizes seen in this study were small (<0.1 cup equivalents/day and <0.1 types/day). However, given the multiple factors influencing dietary intake, numerous modest impacts are likely required to make substantive changes to children’s diets. Finally, while we utilized data from a large, well-characterized national sample of diverse caregivers and their children based on race and ethnicity, our sample is primarily low-income, and our findings may not be generalizable to higher-income populations.

Using data from a large, well-characterized national sample of low-income mothers/caregivers and their children, we found a positive relationship between exposure to a greater variety of vegetables at 9 months of age and both total daily vegetable consumption and the number of different types of vegetables consumed per day in early childhood. While greater exposure to breastfeeding in the first year of life was associated with the number of different types of vegetables consumed per day, it was not associated with the total amount of vegetable intake across early childhood. Evaluation of interventions that target consumption of a greater variety of vegetables by older infants (i.e., around 9 months of age) are warranted to establish causality. At the time of the WIC ITFPS-2 study, breastfeeding women received $11/month for fruits and vegetables and children received $8–9/month as part of the standard food package for which all WIC enrollees were eligible. In 2017, the National Academies of Sciences, Engineering and Medicine recommended that the fruit and vegetable allocation in the WIC food package for breastfeeding women and children be quadrupled [50]. Furthermore, during the COVID-19 pandemic, the American Rescue Plan Act and subsequent legislation required USDA to substantially increase the value of the Cash Value Benefit. The impacts of these policy changes on women’s and children’s vegetable consumption warrants further investigation.

## Figures and Tables

**Table 1:** Weighted sample characteristics for children 13, 24, 36, 48 and 60 months old from the WIC ITFPS-2 ^AB^.

Characteristic	Child Age (in Months)				
	13 (Weighted n = 442,166)	24 (Weighted n = 442,243)	36 (Weighted n = 441,704)	48 (Weighted n = 441,456)	60 (Weighted n = 441,247)
Maternal/caregiver race/ethnicity, %					
Hispanic	46.3	46.6	46.9	46.9	46.9
Non-Hispanic white	28.1	27.8	27.6	27.4	27.1
Non-Hispanic black	19.7	20.2	19.9	20.1	20.4
Non-Hispanic other	5.9	5.4	5.7	5.6	5.7
Maternal/caregiver education, %					
High school or less	62.3	57.8	57.2	57.1	54.5
More than high school	37.7	42.2	42.8	43.0	45.5
Marital status, %					
Married	36.9	36.7	38.7	39.3	39.4
Not married	63.1	63.3	61.3	60.7	60.6
Household federal poverty level (FPL), %					
At or below 75% FPL	52.9	47.9	43.9	44.1	44.6
Between 75–130% FPL	29.9	31.4	32.3	31.5	32.1
Above 130% FPL	17.2	20.8	23.9	24.4	23.3
Household food security, %					
Very low food security	11.7	11.4	9.5	10.9	10.5
Low food security	20.9	16.8	15.7	13.0	12.9
High or marginal food security	67.4	71.8	74.9	76.0	76.7
Current WIC participant, %	85.9	71.1	59.3	52.5	52.7
Child sex female, %	49.1	49.0	47.6	47.9	48.4
Breastfeeding					
Not breastfed ^C^	24.0	23.6	23.5	24.3	24.3
Any breastfeeding at 3 months	61.4	61.2	61.4	59.6	59.7
Any breastfeeding at 6 months	27.8	27.1	26.9	26.5	26.3
Any breastfeeding at 12 months	18.2	17.5	17.0	17.3	17.5
Duration, any (months), mean ± SE	4.0 ± 0.15	3.9 ± 0.15	3.9 ± 0.15	3.8 ± 0.15	3.8 ± 0.15
Introduction to vegetables ^D^					
Age when first introduced (in months), mean ± SE	5.7 ± 0.05	5.7 ± 0.05	5.7 ± 0.05	5.7 ± 0.04	5.6 ± 0.04
Different types eaten at 9 months (count), ^E^ mean ± SE	1.2 ± 0.04	1.1 ± 0.05	1.1 ± 0.05	1.1 ± 0.05	1.1 ± 0.05
Vegetables were first or second food introduced, % yes	76.2	75.3	75.6	75.6	75.5
Total intake of vegetables (cup equivalents/day), mean ± SE	0.6 ± 0.01	0.7 ± 0.01	0.8 ± 0.02	0.8 ± 0.02	0.9 ± 0.02
Different types of vegetables eaten (count), ^E^ mean ± SE	2.2 ± 0.04	2.3 ± 0.05	2.3 ± 0.05	2.3 ± 0.04	2.3 ± 0.04

^A^ Special Supplemental Nutrition Program for Women, Infants, and Children Infant and Toddler Feeding Practices Study 2. ^B^ For characteristics that do not change over time (maternal/caregiver race/ethnicity, child sex, breastfeeding, and introduction to vegetables), numbers may vary slightly due to sample weighting at the respective child ages. Unweighted sample (n = 3773) characteristics can be found in Supplemental Table S2. ^C^ Mothers/caregivers who were not breastfeeding when discharged from the hospital post-birth were set to a breastfeeding duration of 0 days (i.e., Not breastfed). ^D^ Vegetable types include (1) dark green vegetables; (2) tomatoes and tomato products; (3) other red and orange vegetables, excluding tomatoes and tomato products; (4) white potatoes; (5) other starchy vegetables, excluding white potatoes; (6) legumes; and (7) other vegetables not included in the vegetable components 1–6. ^E^ Types of vegetables (score 0–7) was calculated with consumption (>0 cup equivalents) of (1) dark green vegetables; (2) tomatoes and tomato products; (3) other red and orange vegetables, excluding tomatoes and tomato products; (4) white potatoes; (5) other starchy vegetables, excluding white potatoes; (6) legumes; and (7) other vegetables each counting as 1 point.

**Table 2:** Adjusted associations ^A^ between infant breastfeeding exposure and timing of vegetable introduction with total vegetable intake ^B^ (cup equivalents/day) in early childhood.

Characteristic	Total Vegetable Intake (Cup Equivalents on a Given Day)				
	Child Age 13 Months (Weighted n = 411,671)	Child Age 24 Months (Weighted n = 416,377)	Child Age 36 Months (Weighted n = 418,973)	Child Age 48 Months (Weighted n = 419,362)	Child Age 60 Months (Weighted n = 417,723)
	β ± SE (95% CI)				
Breastfeeding					
Not breastfed after hospital discharge vs. ever breastfed	−0.03 ± 0.03 (−0.096, 0.026)	0.02 ± 0.04 (−0.067, 0.106)	0.01 ± 0.04 (−0.070, 0.095)	−0.03 ± 0.05 (−0.118, 0.064)	**−0.08 ± 0.04** (−0.168, −0.002) *
Any breastfeeding at 3 months vs. not breastfed at 3 months	0.06 ± 0.04 (−0.021, 0.141)	−0.02 ± 0.04 (−0.106, 0.076)	−0.03 ± 0.07 (−0.190, 0.129)	−0.05 ± 0.07 (−0.194, 0.089)	**0.14 ± 0.06** (0.017, 0.266) *
Any breastfeeding at 6 months vs. not breastfed at 6 months	0.02 ± 0.04 (−0.054, 0.093)	0.03 ± 0.03 (−0.029, 0.091)	0.02 ± 0.06 (−0.089, 0.139)	−0.05 ± 0.06 (−0.170, 0.061)	0.08 ± 0.08 (−0.084, 0.235)
Any breastfeeding at 12 months vs. not breastfed at 12 months	−0.04 ± 0.04 (−0.112, 0.039)	0.00 ± 0.04 (−0.076, 0.076)	0.02 ± 0.06 (−0.108, 0.140)	−0.02 ± 0.07 (−0.165, 0.117)	0.05 ± 0.07 (−0.090, 0.198)
Duration, any (months), mean ± SE	−0.00 ± 0.00 (−0.007, 0.006)	0.00 ± 0.00 (−0.006, 0.007)	0.00 ± 0.01 (−0.010, 0.012)	−0.00 ± 0.01 (−0.016, 0.007)	0.01 ± 0.01 (−0.007, 0.020)
Introduction to vegetables					
Age when first introduced (in months)	−0.02 ± 0.01 (−0.034, 0.001)	−0.01 ± 0.01 (−0.033, 0.016)	−0.03 ± 0.02 (−0.060, 0.004)	−0.02 ± 0.01 (−0.050, 0.001)	−0.01 ± 0.02 (−0.049, 0.024)
Different types eaten at 9 months (count) ^C^	**0.06 ± 0.01** (0.003, 0.083) *	**0.05 ± 0.02** (0.002, 0.084) *	**0.04 ± 0.02** (0.008, 0.070) *	0.05 ± 0.03 (−0.010, 0.100)	**0.09 ± 0.03** (0.031, 0.145) *
Vegetables were first or second food introduced, yes/no	**0.14 ± 0.04** (0.058, 0.213) *	0.01 ± 0.04 (−0.074, 0.087)	0.02 ± 0.05 (−0.085, 0.127)	0.00 ± 0.06 (−0.118, 0.127)	0.07 ± 0.06 (−0.060, 0.201)

^A^ Data derived from weighted linear and logistic regression models controlling for child-level (sex, WIC participation status), maternal/caregiver-level (race/ethnicity, marital status, education) and household-level (federal poverty level, food security) variables, and accounting for cluster survey design. Breastfeeding models also controlled for age when first introduced to vegetables; Introduction to vegetable models also controlled for breastfeeding duration in months. ^B^ Total vegetables include (1) dark green vegetables; (2) tomatoes and tomato products; (3) other red and orange vegetables, excluding tomatoes and tomato products; (4) white potatoes; (5) other starchy vegetables, excluding white potatoes; (6) legumes; and (7) other vegetables not included in the vegetable components 1–6. ^C^ Types of vegetables (score 0–7) was calculated with consumption (>0 cup equivalents) of (1) dark green vegetables; (2) tomatoes and tomato products; (3) other red and orange vegetables, excluding tomatoes and tomato products; (4) white potatoes; (5) other starchy vegetables, excluding white potatoes; (6) legumes; and (7) other vegetables each counting as 1 point. * Indicates a *p*-value < 0.05.

**Table 3:** Adjusted associations ^A^ between infant breastfeeding exposure and vegetable introduction with the number of different types of vegetables consumed/day ^B^ (count 0–7) in early childhood.

Characteristic	Number of Types of Vegetables Consumed on a Given Day ^B^				
	Child age 13 Months (Weighted n = 411,671)	Child Age 24 Months (Weighted n = 416,377)	Child Age 36 Months (Weighted n = 418,973)	Child Age 48 Months (Weighted n = 419,362)	Child Age 60 Months (Weighted n = 417,723)
	β ± SE (95% CI)				
Breastfeeding					
Not breastfed after hospital discharge vs. ever breastfed	−0.018 ± 0.03 (−0.074, 0.037)	−0.04 ± 0.04 (−0.116, 0.029)	−0.05 ± 0.03 (−0.120, 0.020)	−0.05 ± 0.03 (−0.106, 0.002)	**−0.10 ± 0.04** (−0.190, −0.020) *
Any breastfeeding at 3 months vs. not breastfed at 3 months	**0.10 ± 0.05** (0.001, 0.196) *	**0.22 ± 0.06** (0.095, 0.336) *	0.06 ± 0.07 (−0.069, 0.196)	**0.14 ± 0.05** (0.040, 0.247) *	**0.24 ± 0.07** (0.112, 0.376) *
Any breastfeeding at 6 months vs. not breastfed at 6 months	**0.10 ± 0.04** (0.025, 0.166) *	**0.13 ± 0.04** (0.057, 0.205) *	0.07 ± 0.06 (−0.05, 0.192)	**0.14 ± 0.04** (0.054, 0.225) *	**0.17 ± 0.05** (0.061, 0.274) *
Any breastfeeding at 12 months vs. not breastfed at 12 months	**0.11 ± 0.04** (0.029, 0.198) *	**0.12 ± 0.04** (0.033, 0.209) *	0.01 ± 0.07 (−0.135, 0.150)	**0.13 ± 0.05** (0.026, 0.244) *	**0.15 ± 0.06** (0.030, 0.261) *
Duration, any (months), mean ± SE	**0.01 ± 0.00** (0.006, 0.017) *	**0.01 ± 0.00** (0.006, 0.021) *	0.00 ± 0.01 (−0.010, 0.016)	**0.01 ± 0.00** (0.006, 0.023) *	**0.02 ± 0.00** (0.006, 0.025) *
Introduction to vegetables					
Age when first introduced (in months)	**−0.03 ± 0.01** (−0.057, −0.005) *	−0.01 ± 0.01 (−0.036, 0.009)	−0.01 ± 0.01 (−0.042, 0.018)	−0.02 ± 0.02 (−0.057, 0.014)	**−0.03 ± 0.01** (−0.064, −0.005) *
Different types eaten at 9 months (count) ^B^	0.09 ± 0.01 (0.065, 0.118) *	0.08 ± 0.01 (0.048, 0.106) *	0.06 ± 0.01 (0.036, 0.087) *	0.06 ± 0.01 (0.028, 0.087) *	**0.07 ± 0.02** (0.037, 0.111) *
Vegetables were first or second food introduced, yes/no	0.06 ± 0.04 (−0.016, 0.140)	0.02 ± 0.05 (−0.086, 0.123)	0.04 ± 0.05 (−0.055, 0.143)	0.02 ± 0.04 (−0.068, 0.112)	0.00 ± 0.05 (−0.097, 0.102)

^A^ Data derived from weighted negative binomial regression models controlling for child-level (sex, WIC participation status), maternal/caregiver-level (race/ethnicity, marital status, education) and household-level (income, food security) variables, and accounting for cluster survey design. Breastfeeding models also controlled for age when first introduced to vegetables; introduction to vegetable models also controlled for breastfeeding duration in months. ^B^ Types of vegetables (score 0–7) was calculated with consumption (>0 cup equivalents) of (1) dark green vegetables; (2) tomatoes and tomato products; (3) other red and orange vegetables, excluding tomatoes and tomato products; (4) white potatoes; (5) other starchy vegetables, excluding white potatoes; (6) legumes; and (7) other vegetables each counting as 1 point. * Indicates a *p*-value < 0.05.

## Data Availability

Data used in analysis can be obtained by contacting USDA FNS at OPSDataRequests@usda.gov (accessed on 10 May 2021).

## Supplementary Materials

The following supporting information can be downloaded at: https://www.mdpi.com/article/10.3390/nu14091740/s1, Table S1: Data collection sources and timing for key variables included in analysis; Table S2: Unweighted sample characteristics for children 13, 24, 36, 48 and 60 months old from the WIC ITFPS-2; Table S3. Adjusted associations between infant breastfeeding and vegetable introduction with vegetable intake (by type) in early childhood.
