# Supplementary material for: The Relationship between Breastfeeding and Initial Vegetable Introduction with Vegetable Consumption in a National Cohort of Children Ages 1–5 Years from Low-Income Households
Source: Nutrients. 2022 Apr 22;14(9):1740. doi: 10.3390/nu14091740 (PMC9101240; doi:10.3390/nu14091740)
Supplement: Supplementary file 1 [file nutrients-14-01740-s001.zip › nutrients-1659191-supplementary.pdf]

Supplemental Table S1. Data collection sources and timing for key variables included in analysis.

Variable Type	Variable	Variable Data Source	Timing of Collection (by study child age)
Outcome	Child's vegetable[A] intake (cups equivalents/day)	24-hr dietary recall[B]	Months 13, 24, 36, 48, 60
Outcome/ Predictor	Number of different types of vegetables eaten (count/day)	24-hr dietary recall[B]	Months 9, 13, 24, 36, 48, 60
Predictor	Child's age at introduction to vegetables	Participant interview; Age at which infant/child is first introduced to vegetables	Months 1, 3, 5, 7, 9, 11, 13, 15, 18, 24
Predictor	Is vegetable first or second food introduced	Participant interview; Age at which infant/child is first introduced to vegetables	Months 1, 3, 5, 7, 9, 11, 13, 15, 18, 24
Predictor	Breastfeeding initiation and duration	Participant interview; Whether infant/child is being fed human milk after hospital discharge. If no, infant/child age in days when completely stopped receiving human milk	Months 1, 3, 5, 7, 9, 11, 13
Predictor	Formula initiation and duration	Participant interview; Whether infant/child is being fed formula. If yes, infant/child age in days when started receiving formula	Months 1, 3, 5, 7, 9, 11, 13
Covariate	Child's sex	Participant interview; Sex of child participant	Month 1 or 3 (baseline interview)
Covariate	Child's WIC[B] participation	Participant interview; Whether mother/caregiver is receiving WIC benefits for the study child	Month 13, 24, 36, 48
Covariate	Mother/caregiver's race/ethnicity	Participant interview; Race and Hispanic origin	Month 1 or 3 (baseline interview)
Covariate	Mother/caregiver's education	Participant interview; Highest level of school completed	Month 1, 24, 30, 54
Covariate	Mother/caregiver's marital status	Participant interview; Current marital status	Month 13, 24, 36, 48
Covariate	Household federal poverty level (FPL)	Participant interview; Household income, household size	Month 13, 24, 36, 48
Covariate	Household food security	Participant interview; Household food security score	Month 1, 13, 24, 36, 48

[A] Vegetable types include (1) dark green vegetables; (2) tomatoes and tomato products; (3) other red and orange vegetables, excluding tomatoes and tomato products; (4) white potatoes; (5) other starchy vegetables, excluding white potatoes ; (6) legumes; and (7) other vegetables not included in the vegetable components listed above.

[B]Automated Multi-Pass Method for 24-hr dietary recall.

[C] WIC = Special Supplemental Nutrition Program for Women, Infants, and Children

Supplemental Table S2. Unweighted sample characteristics for children 13, 24, 36, 48 and 60 months old from the WIC ITFPS-2[A].

Characteristic	Child Age (in months)				
	13 (n=3773)	24 (n=3773)	36 (n=3773)	48 (n=3773)	60 (n=3773)
Maternal/caregiver race/ethnicity, %					
Hispanic	38.2	38.2	38.2	38.2	38.2
Non-Hispanic white	31.7	31.7	31.7	31.7	31.7
Non-Hispanic black	24.1	24.1	24.1	24.1	24.1
Non-Hispanic other	6.0	6.0	6.0	6.0	6.0
Maternal/caregiver education, %					
High school or less	62.1	59.7	59.4	59.4	57.2
More than high school	37.9	40.3	40.6	40.6	42.8
Marital status, %					
Married	33.6	33.9	35.9	35.9	35.9
Not married	66.4	66.1	64.1	64.1	64.1
Household federal poverty level (FPL), %					
At or below 75% Federal Poverty Level	55.1	47.8	44.2	44.8	44.8
Between 75-130% Federal Poverty Level	29.1	31.3	31.6	30.2	30.2
Above 130% Federal Poverty Level	15.7	20.9	24.2	25.0	25.0
Household food security, %					
Very low food security	13.1	11.4	11.2	11.8	11.8
Low food security	22.5	17.7	16.5	13.0	13.0
High or marginal food security	64.4	71.0	72.4	75.2	75.2
Current WIC participant, %	88.1	68.4	55.5	50.2	50.2
Child sex female, %	48.9	48.9	48.9	48.9	48.9
Breastfeeding					
Not breastfed after hospital discharge	27.3	27.3	27.3	27.3	27.3
Any breastfeeding at 3 months	52.2	52.2	52.2	52.2	52.2
Any breastfeeding at 6 months	22.4	22.4	22.4	22.4	22.4
Any breastfeeding at 12 months	13.6	13.6	13.6	13.6	13.6
Duration, any (months), mean ± SE	3.29 ± 0.07	3.29 ± 0.07	3.29 ± 0.07	3.29 ± 0.07	3.29 ± 0.07
Introduction to vegetables[B]					
Age when first introduced (in months), mean ± SE	5.62 ± 0.03	5.62 ± 0.03	5.62 ± 0.03	5.62 ± 0.03	5.62 ± 0.03
Different types eaten at 9 months (count),[C] mean ± SE	0.99 ± 0.02	0.99 ± 0.02	0.99 ± 0.02	0.99 ± 0.02	0.99 ± 0.02
Vegetables were first or second food introduced, yes/no	75.4	75.4	75.4	75.4	75.4

Total intake (cup equivalents/day), mean ± SE	0.63 ± 0.61	0.70 ± 0.65	0.82 ± 0.75	0.80 ± 0.73	0.89 ± 0.83
Different types eaten (count),[C] mean ± SE	1.60 ± 1.54	1.51 ± 1.59	1.61 ± 1.59	1.54 ± 1.54	1.53 ± 1.54

[A] Special Supplemental Nutrition Program for Women, Infants, and Children Infant and Toddler Feeding Practices Study 2

[B] Vegetable types include (1) dark green vegetables; (2) tomatoes and tomato products; (3) other red and orange vegetables, excluding tomatoes and tomato products; (4) white potatoes; (5) other starchy vegetables, excluding white potatoes ; (6) legumes; and (7) other vegetables not included in the vegetable components 1-6.

[C] Types of vegetables (score 0-7) was calculated with consumption (>0 cup equivalents) of (1) dark green vegetables; (2) tomatoes and tomato products; (3) other red and orange vegetables, excluding tomatoes and tomato products; (4) white potatoes; (5) other starchy vegetables, excluding white potatoes; (6) legumes; and (7) other vegetables each counting as 1 point.

Supplemental Table S3. Adjusted associations[A] between infant breastfeeding and vegetable introduction with vegetable intake (by type) in early childhood.

**Characteristic**	**Child Age in Months**				
	**13 (weighted n=411,671)**	**24 (weighted n=416,377)**	**36 (weighted n=418,973)**	**48 (weighted n=419,362)**	**60 (weighted n= 417,723)**
	**β ± SE (95% CI)**				
**Dark green vegetable intake (cup equivalents/day)**					
Breastfeeding					
Not breastfed after hospital discharge vs. ever breastfed	0.01 ± 0.01 (-0.007, 0.024)	-0.01 ± 0.01 (-0.028, 0.011)	-0.01 ± 0.01 (-0.033, 0.001)	0.00 ± 0.02 (-0.034, 0.036)	**-0.04 ± 0.01 (-0.057, -0.013)***
Any breastfeeding at 3 months vs. not breastfed at 3 months	-0.00 ± 0.01 (-0.018, 0.016)	0.03 ± 0.02 (-0.007, 0.063)	0.03 ± 0.02 (-0.018, 0.070)	0.03 ± 0.02 (-0.008, 0.060)	0.04 ± 0.03 (-0.027, 0.113)
Any breastfeeding at 6 months vs. not breastfed at 6 months	-0.01 ± 0.01 (-0.022, 0.021)	**0.03 ± 0.01 (0.001, 0.057)***	0.02 ± 0.01 (-0.009, 0.049)	0.01 ± 0.01 (-0.012, 0.037)	0.04 ± 0.03 (-0.023, 0.109)
Any breastfeeding at 12 months vs. not breastfed at 12 months	-0.01 ± 0.01 (-0.023, 0.007)	**0.03 ± 0.02 (0.000, 0.061)***	0.01 ± 0.01 (-0.008, 0.028)	0.03 ± 0.02 (-0.004, 0.062)	0.06 ± 0.04 (-0.022, 0.139)
Duration, any (months), mean ± SE	0.00 ± 0.00 (-0.001, 0.001)	**0.00 ± 0.00 (0.000, 0.005)***	0.00 ± 0.00 (-0.000, 0.003)	0.00 ± 0.00 (-0.000, 0.004)	0.00 ± 0.00 (-0.003, 0.011)
Introduction to vegetables					
Age when first introduced (in months), mean ± SE	-0.00 ± 0.00 (-0.004, 0.003)	-0.00 ± 0.00 (-0.012, 0.004)	-0.00 ± 0.00 (-0.012, 0.003)	-0.00 ± 0.00 (-0.007, 0.005)	0.00 ± 0.00 (-0.007, 0.012)
Different types eaten at 9 months (count),[B] mean ± SE	0.00 ± 0.00 (-0.002, 0.007)	-0.01 ± 0.00 (-0.014, 0.003)	0.00 ± 0.01 (-0.011, 0.014)	**0.01 ± 0.00 (0.003, 0.020)***	0.01 ± 0.01 (-0.005, 0.023)
Vegetables were first or second food introduced, **yes**/no	-0.01 ± 0.01 (-0.026, 0.010)	**0.03 ± 0.01 (0.006, 0.048)***	0.01 ± 0.01 (-0.014, 0.028)	0.01 ± 0.01 (-0.009, 0.038)	0.02 ± 0.02 (-0.015, 0.064)
**Tomatoes and tomato product intake (cup equivalents/day)**					
Breastfeeding					
Not breastfed after hospital discharge vs. ever breastfed	-0.01 ± 0.01 (-0.031, 0.009)	0.01 ± 0.02 (-0.031, 0.054)	-0.01 ± 0.02 (-0.049, 0.026)	-0.01 ± 0.02 (-0.039, 0.027)	0.01 ± 0.02 (-0.024, 0.045)
Any breastfeeding at 3 months vs. not breastfed at 3 months	-0.01 ± 0.01 (-0.036, 0.021)	-0.03 ± 0.03 (-0.079, 0.024)	0.03 ± 0.03 (-0.021, 0.088)	-0.03 ± 0.03 (-0.090, 0.073)	-0.02 ± 0.03 (-0.074, 0.043)
Any breastfeeding at 6 months vs. not breastfed at 6 months	-0.01 ± 0.01 (-0.026, 0.006)	0.00 ± 0.02 (-0.028, 0.037)	0.01 ± 0.02 (-0.028, 0.055)	**-0.05 ± 0.02 (-0.092, -0.010)***	-0.01 ± 0.02 (-0.058, 0.036)

Any breastfeeding at 12 months vs. not breastfed at 12 months	-0.01 ± 0.01 (-0.026, 0.007)	-0.01 ± 0.01 (-0.037, 0.020)	-0.01 ± 0.03 (-0.061, 0.041)	**-0.06 ± 0.02 (-0.096, -0.020)***	-0.00 ± 0.03 (-0.061, 0.061)
Duration, any (months), mean ± SE	-0.00 ± 0.00 (-0.003, 0.002)	-0.00 ± 0.00 (-0.003, 0.002)	0.00 ± 0.00 (-0.003, 0.006)	-0.00 ± 0.00 (-0.008, 0.000)	-0.00 ± 0.00 (-0.005, 0.004)
Introduction to vegetables					
Age when first introduced (in months), mean ± SE	-0.00 ± 0.00 (-0.009, 0.002)	0.00 ± 0.01 (-0.015, 0.007)	0.00 ± 0.01 (-0.014, 0.011)	**-0.02 ± 0.01 (-0.030, -0.003)***	0.00 ± 0.01 (-0.017, 0.020)
Different types eaten at 9 months (count),[B] mean ± SE	0.01 ± 0.00 (-0.004, 0.016)	-0.00 ± 0.01 (-0.014, 0.010)	0.00 ± 0.01 (-0.021, 0.018)	0.01 ± 0.01 (-0.015, 0.033)	0.00 ± 0.01 (-0.014, 0.018)
Vegetables were first or second food introduced, **yes**/no	0.01 ± 0.01 (-0.012, 0.038)	0.03 ± 0.02 (-0.008, 0.062)	0.01 ± 0.02 (-0.020, 0.049)	-0.01 ± 0.03 (-0.064, 0.046)	0.04 ± 0.02 (-0.005, 0.080)
**Other red and orange vegetables, excluding tomatoes and tomato products, intake (cup equivalents/day)**					
Breastfeeding					
Not breastfed after hospital discharge vs. ever breastfed	0.00 ± 0.01 (-0.028, 0.027)	-0.01 ± 0.01 (-0.024, 0.005)	**-0.02 ± 0.01 (-0.033, -0.002)***	-0.01 ± 0.01 (-0.026, 0.010)	**-0.02 ± 0.01 (-0.039, -0.005)***
Any breastfeeding at 3 months vs. not breastfed at 3 months	0.01 ± 0.02 (-0.033, 0.052)	**0.02 ± 0.01 (0.006, 0.037)***	-0.01 ± 0.02 (-0.041, 0.038)	-0.02 ± 0.03 (-0.080, 0.045)	0.03 ± 0.01 (-0.002, 0.059)
Any breastfeeding at 6 months vs. not breastfed at 6 months	0.02 ± 0.02 (-0.011, 0.057)	0.00 ± 0.01 (-0.014, 0.017)	0.01 ± 0.01 (-0.015, 0.041)	0.00 ± 0.01 (-0.020, 0.028)	0.02 ± 0.01 (-0.007, 0.047)
Any breastfeeding at 12 months vs. not breastfed at 12 months	0.02 ± 0.01 (-0.013, 0.045)	-0.01 ± 0.01 (-0.024, 0.008)	0.02 ± 0.02 (-0.010, 0.054)	-0.00 ± 0.01 (-0.026, 0.023)	0.01 ± 0.01 (-0.010, 0.040)
Duration, any (months), mean ± SE	0.00 ± 0.00 (-0.001, 0.005)	0.00 ± 0.00 (-0.001, 0.001)	0.00 ± 0.00 (-0.002, 0.004)	-0.00 ± 0.00 (-0.003, 0.002)	0.00 ± 0.00 (-0.001, 0.004)
Introduction to vegetables					
Age when first introduced (in months), mean ± SE	-0.00 ± 0.00 (-0.007, 0.007)	0.00 ± 0.00 (-0.007, 0.002)	**-0.01 ± 0.00 (-0.011, -0.002)***	-0.00 ± 0.00 (-0.008, 0.003)	-0.01 ± 0.00 (-0.011, 0.000)
Different types eaten at 9 months (count),[B] mean ± SE	0.00 ± 0.01 (-0.009, 0.018)	0.00 ± 0.00 (-0.003, 0.008)	0.00 ± 0.00 (-0.008, 0.010)	0.00 ± 0.01 (-0.012, 0.021)	**0.02 ± 0.01 (0.005, 0.037)***
Vegetables were first or second food introduced, **yes**/no	**0.04 ± 0.01 (0.012, 0.063)***	0.01 ± 0.01 (-0.002, 0.029)	0.02 ± 0.01 (-0.002, 0.039)	0.02 ± 0.01 (-0.005, 0.048)	0.01 ± 0.01 (-0.013, 0.042)
**White potatoes intake (cup equivalents/day)**					
Breastfeeding					
Not breastfed after hospital discharge vs. ever breastfed	0.01 ± 0.02 (-0.029, 0.043)	0.03 ± 0.03 (-0.021, 0.086)	**0.07 ± 0.03 (0.010, 0.129)***	0.03 ± 0.02 (-0.011, 0.063)	0.02 ± 0.02 (-0.016, 0.069)

Any breastfeeding at 3 months vs. not breastfed at 3 months	0.01 ± 0.02 (-0.034, 0.057)	**-0.08 ± 0.03 (-0.138, -0.021)***	**-0.10 ± 0.03 (-0.164, -0.038)***	**-0.106 ± 0.03 (-0.170, -0.041)***	-0.05 ± 0.04 (-0.128, 0.037)
Any breastfeeding at 6 months vs. not breastfed at 6 months	-0.00 ± 0.02 (-0.035, 0.033)	**-0.04 ± 0.02 (-0.082, -0.008)***	-0.05 ± 0.03 (-0.106, 0.005)	-0.05 ± 0.04 (-0.126, 0.024)	-0.07 ± 0.04 (-0.137, 0.006)
Any breastfeeding at 12 months vs. not breastfed at 12 months	-0.03 ± 0.02 (-0.069, 0.010)	-0.05 ± 0.03 (-0.098, 0.005)	-0.04 ± 0.03 (-0.098, 0.014)	-0.05 ± 0.04 (-0.130, 0.029)	-0.04 ± 0.03 (-0.105, 0.031)
Duration, any (months), mean ± SE	-0.00 ± 0.00 (-0.005, 0.001)	**-0.01 ± 0.00 (-0.009, -0.001)***	**-0.01 ± 0.00 (-0.011, -0.001)***	-0.01 ± 0.00 (-0.014, 0.001)	-0.01 ± 0.00 (-0.013, 0.000)
Introduction to vegetables					
Age when first introduced (in months), mean ± SE	-0.01 ± 0.01 (-0.024, 0.005)	0.00 ± 0.00 (-0.008, 0.012)	-0.02 ± 0.01 (-0.034, 0.003)	0.00 ± 0.01 (-0.011, 0.017)	-0.01 ± 0.01 (-0.020, 0.010)
Different types eaten at 9 months (count),[B] mean ± SE	0.01 ± 0.01 (-0.008, 0.021)	0.02 ± 0.01 (-0.003, 0.038)	0.00 ± 0.01 (-0.02, 0.025)	0.00 ± 0.02 (-0.030, 0.033)	0.01 ± 0.01 (-0.017, 0.040)
Vegetables were first or second food introduced, **yes**/no	0.03 ± 0.02 (-0.009, 0.066)	-0.04 ± 0.03 (-0.091, 0.012)	-0.03 ± 0.02 (-0.083, 0.016)	-0.03 ± 0.03 (-0.081, 0.028)	0.01 ± 0.03 (-0.053, 0.074)
**Other starchy vegetables, excluding white potatoes, intake (cup equivalents/day)**					
Breastfeeding					
Not breastfed after hospital discharge vs. ever breastfed	-0.01 ± 0.01 (-0.027, 0.006)	0.01 ± 0.01 (-0.011, 0.0258)	0.00 ± 0.01 (-0.018, 0.019)	-0.00 ± 0.01 (-0.022, 0.017)	-0.01 ± 0.01 (-0.033, 0.005)
Any breastfeeding at 3 months vs. not breastfed at 3 months	0.01 ± 0.01 (-0.014, 0.038)	-0.02 ± 0.02 (-0.058, 0.008)	0.00 ± 0.02 (-0.028, 0.033)	**0.04 ± 0.01 (0.006, 0.065)***	0.02 ± 0.01 (-0.010, 0.050)
Any breastfeeding at 6 months vs. not breastfed at 6 months	-0.00 ± 0.01 (-0.019, 0.019)	-0.01 ± 0.01 (-0.033, 0.009)	-0.01 ± 0.01 (-0.034, 0.016)	0.01 ± 0.01 (-0.012, 0.038)	0.02 ± 0.01 (-0.007, 0.038)
Any breastfeeding at 12 months vs. not breastfed at 12 months	-0.00 ± 0.01 (-0.022, 0.020)	-0.01 ± 0.01 (-0.034, 0.020)	0.00 ± 0.01 (-0.028, 0.028)	0.03 ± 0.02 (-0.005, 0.057)	0.01 ± 0.02 (-0.020, 0.041)
Duration, any (months), mean ± SE	-0.00 ± 0.00 (-0.002, 0.002)	0.00 ± 0.00 (-0.003, 0.001)	-0.00 ± 0.00 (-0.003, 0.002)	0.00 ± 0.00 (-0.001, 0.005)	0.00 ± 0.00 (-0.001, 0.003)
Introduction to vegetables					
Age when first introduced (in months), mean ± SE	0.00 ± 0.00 (-0.009, 0.002)	0.00 ± 0.00 (-0.006, 0.008)	0.00 ± 0.00 (-0.005, 0.005)	-0.01 ± 0.00 (-0.015, 0.001)	0.01 ± 0.01 (-0.005, 0.029)
Different types eaten at 9 months (count),[B] mean ± SE	**0.01 ± 0.00 (0.002, 0.017)***	0.01 ± 0.00 (-0.002, 0.014)	0.01 ± 0.00 (-0.002, 0.012)	0.00 ± 0.01 (-0.009, 0.014)	0.01 ± 0.01 (-0.007, 0.017)
Vegetables were first or second food introduced, **yes**/no	0.02 ± 0.01 (-0.002, 0.039)	**-0.03 ± 0.01 (-0.051, -0.002)***	-0.01 ± 0.01 (-0.036, 0.019)	-0.02 ± 0.03 (-0.07, 0.032)	0.01 ± 0.02 (-0.025, 0.044)
**Legumes intake (cup equivalents/day)**					

Breastfeeding					
Not breastfed after hospital discharge vs. ever breastfed	-0.01 ± 0.01 (-0.023, 0.006)	-0.01 ± 0.01 (-0.031, 0.021)	-0.01 ± 0.01 (-0.03, 0.009)	**-0.02 ± 0.01 (-0.043, -0.001)***	0.01 ± 0.01 (-0.020, 0.035)
Any breastfeeding at 3 months vs. not breastfed at 3 months	**0.01 ± 0.01 (0.002, 0.027)***	0.00 ± 0.02 (-0.04, 0.048)	0.01 ± 0.02 (-0.035, 0.052)	0.03 ± 0.02 (-0.013, 0.064)	0.01 ± 0.02 (-0.040, 0.059)
Any breastfeeding at 6 months vs. not breastfed at 6 months	0.01 ± 0.01 (-0.010, 0.034)	0.01 ± 0.01 (-0.021, 0.037)	0.03 ± 0.02 (-0.005, 0.065)	0.01 ± 0.01 (-0.021, 0.039)	0.03 ± 0.02 (-0.011, 0.073)
Any breastfeeding at 12 months vs. not breastfed at 12 months	0.01 ± 0.01 (-0.015, 0.029)	0.01 ± 0.01 (-0.021, 0.035)	0.03 ± 0.02 (-0.01, 0.076)	0.01 ± 0.02 (-0.026, 0.037)	0.01 ± 0.02 (-0.035, 0.061)
Duration, any (months), mean ± SE	0.00 ± 0.00 (-0.001, 0.003)	0.00 ± 0.00 (-0.002, 0.004)	0.00 ± 0.00 (-0.001, 0.006)	0.00 ± 0.00 (-0.002, 0.003)	0.00 ± 0.00 (-0.001, 0.006)
Introduction to vegetables					
Age when first introduced (in months), mean ± SE	0.00 ± 0.00 (-0.005, 0.004)	0.00 ± 0.00 (-0.009, 0.005)	**0.02 ± 0.01 (0.003, 0.028)***	0.00 ± 0.00 (-0.006, 0.011)	-0.01 ± 0.00 (-0.017, 0.001)
Different types eaten at 9 months (count),[B] mean ± SE	0.00 ± 0.01 (-0.006, 0.015)	0.01 ± 0.01 (-0.005, 0.032)	-0.00 ± 0.01 (-0.013, 0.013)	-0.00 ± 0.01 (-0.016, 0.011)	0.01 ± 0.01 (-0.010, 0.023)
Vegetables were first or second food introduced, **yes**/no	0.01 ± 0.01 (-0.009, 0.036)	0.01 ± 0.01 (-0.015, 0.025)	-0.01 ± 0.02 (-0.037, 0.026)	0.01 ± 0.01 (-0.02, 0.035)	-0.03 ± 0.03 (-0.082, 0.027)
**Other vegetables intake (cup equivalents/day)**					
Breastfeeding					
Not breastfed after hospital discharge vs. ever breastfed	-0.02 ± 0.01 (-0.041, 0.003)	-0.01 ± 0.02 (-0.054, 0.036)	-0.00 ± 0.02 (-0.039, 0.033)	-0.02 ± 0.02 (-0.050, 0.020)	**-0.06 ± 0.01 (-0.087, -0.027)***
Any breastfeeding at 3 months vs. not breastfed at 3 months	0.02 ± 0.02 (-0.018, 0.063)	**0.06 ± 0.03 (0.010, 0.116)***	0.00 ± 0.04 (-0.074, 0.078)	0.01 ± 0.03 (-0.040, 0.063)	**0.10 ± 0.03 (0.032, 0.169)***
Any breastfeeding at 6 months vs. not breastfed at 6 months	0.00 ± 0.02 (-0.028, 0.034)	**0.05 ± 0.02 (0.003, 0.088)***	0.01 ± 0.02 (-0.036, 0.052)	0.01 ± 0.02 (-0.037, 0.056)	0.04 ± 0.03 (-0.024, 0.107)
Any breastfeeding at 12 months vs. not breastfed at 12 months	-0.01 ± 0.02 (-0.045, 0.021)	0.03 ± 0.02 (-0.017, 0.083)	0.00 ± 0.03 (-0.056, 0.064)	0.03 ± 0.03 (-0.039, 0.090)	-0.01 ± 0.03 (-0.061, 0.049)
Duration, any (months), mean ± SE	-0.00 ± 0.00 (-0.003, 0.003)	0.00 ± 0.00 (-0.000, 0.008)	0.00 ± 0.00 (-0.004, 0.005)	0.00 ± 0.00 (-0.003, 0.006)	0.00 ± 0.00 (-0.001, 0.008)
Introduction to vegetables					
Age when first introduced (in months), mean ± SE	0.00 ± 0.00 (-0.007, 0.007)	0.00 ± 0.01 (-0.008, 0.012)	**-0.02 ± 0.01 (-0.028, -0.002)***	-0.00 ± 0.01 (-0.015, 0.009)	-0.01 ± 0.01 (-0.028, 0.010)
Different types eaten at 9 months (count),[B] mean ± SE	**0.02 ± 0.00 (0.016, 0.032)***	**0.02 ± 0.01 (0.009, 0.033)***	**0.03 ± 0.01 (0.013, 0.039)***	**0.02 ± 0.01 (0.007, 0.033)***	**0.03 ± 0.01 (0.008, 0.057)***

Vegetables were first or second food introduced, **yes**/no	**0.03 ± 0.01 (0.005, 0.060)***	0.00 ± 0.02 (-0.032, 0.033)	0.03 ± 0.02 (-0.015, 0.073)	0.02 ± 0.02 (-0.021, 0.057)	0.00 ± 0.04 (-0.071, 0.075)

[A] Data derived from weighted linear and logistic regression models controlling for child-level (sex, WIC participation status), maternal/caregiver-level (race/ethnicity, marital status, education) and household-level (federal poverty level, food security) variables, and accounting for cluster survey design. Breastfeeding models also controlled for age when first introduced to vegetables; Introduction to vegetable models also controlled for breastfeeding duration in months.

[B] Types of vegetables (score 0-7) was calculated with consumption (>0 cup equivalents) of (1) dark green vegetables; (2) tomatoes and tomato products; (3) other red and orange vegetables, excluding tomatoes and tomato products; (4) white potatoes; (5) other starchy vegetables, excluding white potatoes; (6) legumes; and (7) other vegetables each counting as 1 point.
